# Serum Bisphenol A Level in Boys with Cryptorchidism: A Step to Male Infertility?

**DOI:** 10.1155/2015/973154

**Published:** 2015-09-28

**Authors:** Marta Diana Komarowska, Adam Hermanowicz, Urszula Czyzewska, Robert Milewski, Ewa Matuszczak, Wojciech Miltyk, Wojciech Debek

**Affiliations:** ^1^Department of Pediatric Surgery, Medical University of Bialystok, Ulica Waszyngtona 17, 15-274 Bialystok, Poland; ^2^Department of Pharmaceutical Analysis, Medical University of Bialystok, Ulica Adama Mickiewicza 2D, 15-222 Bialystok, Poland; ^3^Department of Statistics and Medical Informatics, Medical University of Bialystok, Szpitalna 37, 15-295 Bialystok, Poland

## Abstract

Cryptorchidism is the most common congenital birth defect in boys and affects about 2–4% full-term male neonates. Its etiology is multifactorial. *Purpose*. To evaluate the serum bisphenol A (BPA) levels in boys with cryptorchidism and healthy boys and to assess the risk of environmental exposure to BPA using the authors' questionnaire. The data were acquired from a study on boys with cryptorchidism (*n* = 98) and a control group (*n* = 57). Prior to surgery, all patients had BPA serum levels evaluated. The size, position, rigidity of the testis, and abnormality of the epididymis of the undescended testis were assessed. Parents also completed a questionnaire on the risks of exposure to BPA in everyday life. *Results*. The testes in both groups were similar in size. The turgor of the undescended testis in the group of boys with cryptorchidism was decreased. Free serum BPA level in cryptorchid boys and in the control group was not statistically significant (*p* > 0.05). The conjugated serum BPA level in cryptorchid boys and in the control group was statistically significant (*p* ≤ 0.05). Total serum BPA level in cryptorchid boys and in the control group was statistically significant (*p* < 0.05). Serum total BPA level was related with a positive answer about problems with conception (*p* < 0.02). *Conclusion*. Our study indicated that high serum BPA was associated with cryptorchidism.

## 1. Introduction

Cryptorchidism is the most common congenital birth defect in boys and affects about 2–4% full-term male neonates [1]. Interestingly some epidemiological studies suggest that its prevalence has increased in some countries, for example, Denmark (9.0%) or the United Kingdom (5.9%) [2, 3]. It is much more frequent in low birth weight infants and is diagnosed even in 1/3 of extremely low birth weight infants [4]. It is commonly improperly considered a mild congenital malformation, while it is the best-known risk factor for testicular cancer and reduced fertility in adulthood. The etiology of this disorder remains not fully known [5] and can occur as an isolated disorder or may be associated with other congenital defects. According to the latest research/knowledge, its etiology is multifactorial (genetic, hormonal, mechanical, and environmental) [6, 7]. Environmental factors acting as endocrine disruptors of testicular descent have been suggested as contributing to cryptorchidism and its increased incidence in recent years.

There is great evidence that humans are exposed to ubiquitous endocrine disrupting chemicals (EDCs) due to the rapid development of civilization and industry in recent years [8]. EDCs are exogenous chemicals or mixture of chemicals, which interfere with any aspect of hormone activity [9] and include many different substances, such as xenoestrogens (industrial chemicals), synthetic and natural hormones, and phyto- and mycoestrogens [10]. EDCs, as drugs, natural products, and manufactured chemicals, are present in pesticides, textiles, flame retardants, plastics, fragrances, lotions, paints, toiletries, food, and other products [11].

These substances are similar in structure and function to physiological hormones and may interfere with physiological hormone binding to their receptors [9]. They may bind to all types of receptors (membrane-bound, cytoplasmic, and nuclear receptors). Furthermore, EDCs interfere by altering the production, metabolism, transportation, release, elimination, and binding ability of endogenous hormones.

Bisphenol A (2,2-bis(4-hydroxyphenyl)propane; BPA), a monomer of polycarbonate plastics, is one of the most common endocrine disrupting chemicals [12]. It was first developed as synthetic estrogen (also called xenoestrogen, from the Greek* xeno*, foreign) in the 1890s. Thereafter, BPA has been used in numerous consumer products, including food and water containers, baby bottles, metal can linings, dental filings, and medical tubes [13]. It is used in the production of polycarbonates and epoxy resins, which are widely used in industry.

Therefore, exposure to BPA in the environment is ubiquitous. It is well absorbed from the gastrointestinal tract and through the skin. What is worth emphasizing is that at high temperatures polymers in canned foods, plastic containers, or polycarbonate bottles hydrolyze, and bisphenol A is released [14]. Most BPA was found in the urine as glucuronide conjugate. In humans, free active unconjugated BPA (uBPA) is metabolized by rapid glucuro- or sulfo-conjugation and eliminated via renal clearance [15]. In Europe and the United States, the tolerated daily dose of BPA should not exceed “50 *μ*g/kg/day” [16]. BPA has been found to be a “weak” inducer of estrogenic activity [17]. It can bind to estrogen receptors ER*α* and ER*β* and has a higher affinity for ER*β* in target cells [18].

In our earlier study, we found expression of ER*α* and ER*β* in the mesothelial layer, stromal cells, and the endothelial layer of paratesticular tissues of normal and undescended testes [19]. Furthermore, research shows that BPA estrogenic potency is equal to 17-beta estradiol (E2) for responses mediated by nonnuclear estrogen receptors [20]. On the other hand, BPA can have an antiestrogen effect, by blocking the estrogenic response by competing with endogenous E2 [21, 22]. In higher doses than for estrogen responsive mechanism, BPA can also bind to androgen receptors and can block endogenous androgen action [23].

The aim of the study was to evaluate serum BPA levels in boys with cryptorchidism and healthy boys and to assess the risk of environmental exposure to BPA using the authors' questionnaire.

## 2. Materials and Methods

### 2.1. Ethics

The study was approved by the local ethics committee. Written informed consent was obtained from all parents of the enrolled children.

The data were acquired from a prospective study of boys with cryptorchidism and a control group (boys of the same age, without pathology of the testes) admitted to the Department of Pediatric Surgery, Medical University of Bialystok, Poland, in 2013-2014. All patients lived in the northeast part of the country called the Green Lungs of Poland, which is not industrialized nor polluted. The serum samples, clinical examination, and questionnaire data were collected from all the patients. In all the samples, the routine laboratory examinations were done to exclude inflammation: blood cell count and CRP level.

*The studied group* comprised *n* = 98 boys aged 1–4 years (mean = 27 months) with unilateral congenital cryptorchidism. The control group comprised *n* = 57 healthy boys, without any testicular pathology, aged 1–4 years (mean = 27 months), and admitted for planned inguinal hernia repairs.

All boys underwent surgical procedure. Prior to the operation, all patients had BPA serum levels evaluated. The size, position, rigidity of the testis, and abnormality of the epididymis of the undescended testis were assessed during operation. 5 mL blood samples were taken in the morning between 6 and 7 a.m. on the day of surgery. The blood was centrifuged and the plasma was stored in a BPA free test tube and frozen to −80°C. Serum BPA levels were measured. Parents also completed a questionnaire on the risks of exposure to BPA in everyday life. It was divided into two parts. The first part was about basic data (age, weight, height, place of birth, previous diseases, hormonal therapy, and X-ray exams). The second part was about the environment, daily dietary habits, problems with conception, and pregnancy.

### 2.2. Serum BPA Level

#### 2.2.1. Materials and Chemicals

BPA (99.9%), BPAd_16_ (98%), *β*-glucuronidase (from* Escherichia coli*), pyridine (99.8%), silylation reagent N,O-bis(trimethylsilyl)trifluoroacetamide (BSTFA) with 1% trimethylchlorosilane (TMSC) (99%), ammonium acetate (BioXtra ≥ 98%), acetic acid (ReagentPlus ≥ 99%), acetonitrile (anhydrous, 99.8%), and water (Chromasolv, HPLC grade) were purchased from Sigma-Aldrich (Steinheim, Germany). Chloroform (99%, GC grade) was obtained from J. T. Baker (Gliwice, Poland). Individual stock solutions of BPA and BPAd_16_ at concentration 10 mg/L were dissolved in acetonitrile and stored at −20°C.

#### 2.2.2. Sample Pretreatment Procedure

*Free BPA Levels*. Serum samples were defrosted at room temperature and vortexed. Then 200 *μ*L of aliquots was transferred into a glass tube with a Teflon-coated screw cap and fortified with 5 ng of BPAd_16_. 150 *μ*L of chloroform and 50 *μ*L of acetonitrile were added to each sample. The tubes were closed and shaken by vortex for a few seconds. Then samples were sonicated for 1 min and centrifuged at 5000 rpm and 4°C to allow for a clear phase separation. Finally, 100 *μ*L of the lower organic layer was transferred into a vial. The extracts were evaporated to dryness using a vacuum concentrator and 50 *μ*L of pyridine and 50 *μ*L of BSTFA were added to each sample for derivatization prior to Gas Chromatography (GC-MS) analysis.

*Total BPA Levels*. Serum samples were defrosted at room temperature and vortexed. Then 200 *μ*L of aliquots was transferred into a glass tube with a Teflon-coated screw cap and fortified with 5 ng of BPAd_16_ and 30 *μ*L of *β*-glucuronidase solution (2000 IU in 1 M ammonium acetate buffer, pH = 6.1). The samples were incubated overnight at 37°C. A mixture of 150 *μ*L of chloroform and 50 *μ*L of acetonitrile was added to each sample. The tubes were closed and shaken by vortex for a few seconds. Then samples were sonicated for 1 min and centrifuged at 5000 rpm and 4°C to allow for a clear phase separation. Finally, 100 *μ*L of the lower organic layer was transferred into a vial. The extracts were evaporated to dryness using a vacuum concentrator and 50 *μ*L of pyridine and 50 *μ*L of BSTFA were added to each sample for derivatization prior to GC-MS analysis. 

*GC-MS Analysis*. GC-MS analyses were performed on an Agilent Technologies 5970C VL quadrupole mass spectrometer connected directly to an Agilent Technologies 7890A gas chromatograph and to an autosampler 7693 (Agilent Technologies, Wilmington, DE, USA). Samples were separated on 30 m × 0.25 mm i.d., 0.25 *μ*m film thickness, HP-5MS capillary column J&W (Agilent Technologies, Wilmington, DE, USA). The column temperature was initially set at 130°C and then the temperature was raised to 300°C, at rate of 10°C/min. Ultrapure helium with inline oxygen and moisture trap was used as a carrier gas at a flow rate of 1.0 mL/min. Aliquots of 1 *μ*L were injected in the splitless mode. The injector was kept at 280°C; MS source and MS quad temperatures were 230°C and 150°C, respectively. The MSD was operated with electron impact ionization in selected ion monitoring (SIM) mode. Solvent delay time was set to 8 min. The following (*m/z*) ions were acquired for BPA (357, 358) and BPA_16_ (368, 38). Mass Hunter 6.0.B software was used for instrument control, data acquisition and evaluation (integration, quantification).

### 2.3. Statistical Analysis

Statistical analyses were carried out using Statistica 10.0 StatSoft. To analyze the questionnaire data, we used the chi-square test and Pearson's correlation. The Mann-Whitney *U* test was used to compare the groups. *p* values less than 0.05 were considered significant.

## 3. Results

The testes in both groups were of similar size. There was no statistical significance in the size of the testes. The turgor of the undescended testes in the group of boys with cryptorchidism was decreased in 24 cases (24.5%). The epididymides in 25 cases (25.5%) of undescended testes were separated from the testis. In 14 cases (14%) of boys from the studied group, the testes were impalpable. In the other cases, the testes were found in the inguinal canals.

**Figure 1:**
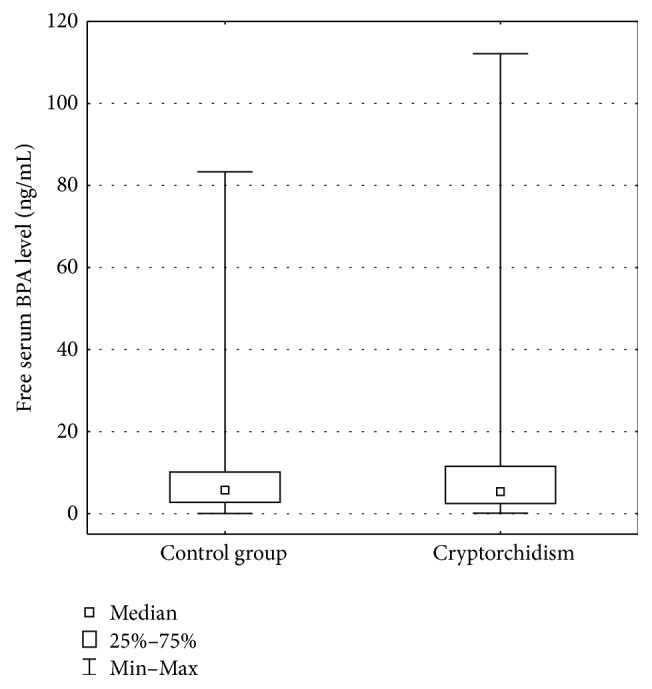
Free serum BPA level (ng/mL).

Free and conjugated BPA levels were assessed in all samples. Free serum BPA levels in cryptorchid boys ranged from 0.11 ng/mL to 112.1 ng/mL (mean 5.3 ng/mL). In the control group, free serum BPA levels ranged from 0.05 ng/mL to 83.4 ng/mL (mean 5.7 ng/mL). The difference was not statistically significant (*p* > 0.05), Figure 1.

**Figure 2:**
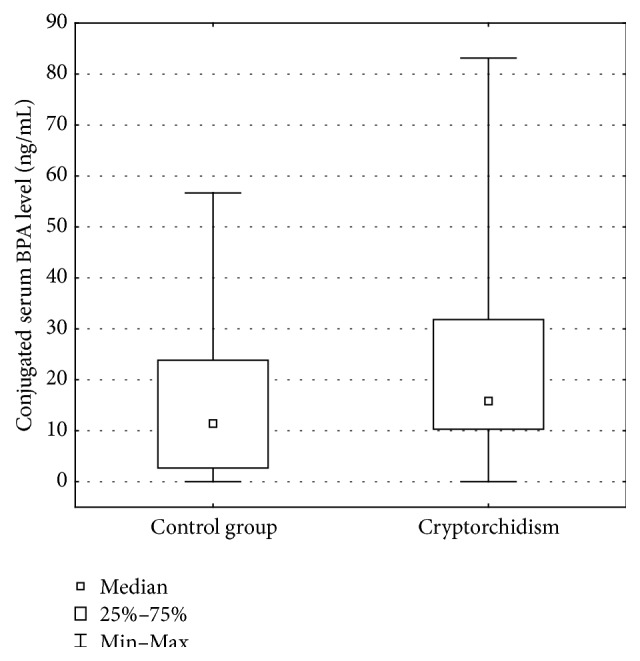
Conjugated serum BPA level (ng/mL).

Conjugated serum BPA levels in cryptorchid boys ranged from 0.0 ng/mL to 83.1 ng/mL (mean 15.8 ng/mL). In the control group, free serum BPA levels ranged from 0.0 ng/mL to 56.7 ng/mL (mean 11.4 ng/mL). The difference was statistically significant (*p* ≤ 0.05) (Figure 2).

**Figure 3:**
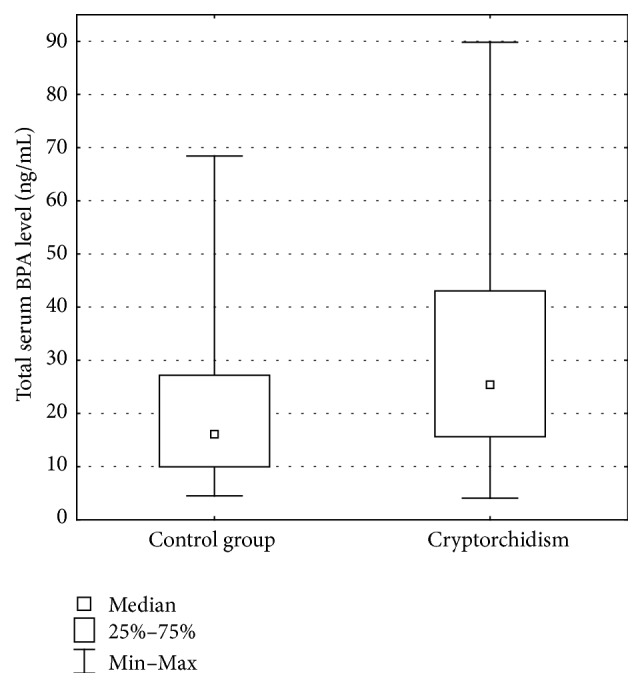
Total serum BPA level (ng/mL).

Total serum BPA levels in cryptorchid boys ranged from 4.1 ng/mL to 89.8 ng/mL (mean 25.4 ng/mL). Total serum BPA levels in the control group ranged from 4.5 ng/mL to 68.5 ng/mL (mean 16.1 ng/mL). The difference was statistically significant (*p* < 0.05) (Figure 3) (Table 1).

We have not found any correlation between the groups and any forms of BPA serum levels in boys and parental smoking, X-ray examinations, hormonal treatment in parents and children, neoplastic diseases, and particularly testicular cancer. The clinical and epidemiologic data from the questionnaire are presented in Table 2.

The serum total BPA level was related to a positive answer about problems with conception. The parents of cryptorchid boys with higher total BPA more frequently reported problems with conception. The difference was statistically significant (*p* < 0.02).

The morphology (cohesion, separation of the epididymis) and localization of the undescended testes were taken into consideration and compared with serum BPA levels. We found no statistically significant correlation in these parameters.

## 4. Discussion

The male reproductive system is regulated by a number of hormones and paracrine factors [24]. Any external factor that impacts the fetus during critical stages of gubernaculum development and testicular descent may increase the risk of cryptorchidism, particularly in genetically susceptible individuals [25]. Endocrine disrupting chemicals could be cofactors in the occurrence of congenital undescended testis.

Recent studies have shown that prenatal exposure to elevated concentrations of BPA causes increased risk of lower birth weight and smaller size for gestational age (SGA), especially in male infants [26]. They are also proven risk factors for cryptorchidism [27]. On the other hand, Lee et al. indicated that high maternal BPA levels were associated with increased birth weight and maternal BPA level had a significantly higher impact on male infants [28]. Gender differences in serum BPA concentration and this phenomenon are probably associated with the variation in the androgen-related metabolism of BPA [29].

A prospective birth cohort study from Denmark and Finland has shown that infertility treatment by intrauterine insemination was associated with an increased risk of cryptorchidism in the male offspring [30], while no association was found in mothers who had intracytoplasmic sperm injection (ICSI) or in vitro fertilization (IVF) treatment. This may be due to different hormonal treatment regimens. In both countries in most cases, intrauterine treatment with clomiphene citrate was used. This drug has estrogenic and antiestrogenic activity [31] and has a relatively long half-life, up to one month. It can affect critical stages of fetal development in the first trimester.

In our study, we did not find any correlation between hormonal treatment and cryptorchidism. The parents of cryptorchid boys in our survey reported that they had problems with insemination. The difference was statistically significant.

According to studies, human fetuses can be very sensitive to exogenous hormones [32, 33], and low-dose exposure during fetal life can result in long-standing adverse effects even after the EDCs are eliminated from the body [34]. This leads to impaired Sertoli cell function resulting in reduced semen quality and testicular germ cell cancer. Leydig cell dysfunction causes cryptorchidism and hypospadias. It was found that fetal Leydig cell differentiation processes are regulated by Sertoli cells [35]. Failures during formation and maturation of the Sertoli cells caused by exposure to endogenous hormonal factors and xenobiotics are related to spermatogenesis failure and are among the crucial factors for Testicular Dysgenesis Syndrome (TDS) [35]. In rodent experimental models, high doses of phthalates alter Leydig cell function affected INSL3 expression and androgen biosynthesis and caused cryptorchidism in rats [36]. Additionally, the toxicokinetics of BPA could be different in children compared with adults. Recent studies suggested that exposure to BPA in newborn is 3–10 times higher than in adults [37, 38].

Our study had some limitations. We used only a single blood sample to measure serum BPA levels. For future investigations, it would be helpful to obtain several blood samples: immediately after birth and at different age before the operation. Presumably the assessment of BPA level in mothers would be helpful also. Because of its short biological half-life, a single blood BPA sample reflects only short-term, but not chronic, exposure. Once administered orally, BPA is very rapidly metabolized with a biological half-life of approximately 6 hours. Expulsion of bisphenol A from the body takes about 24 hours [39], whereas some studies suggest that BPA has a longer half-life and could deposit in fat tissue [40].

On the other hand in studies comparing serum BPA levels in breast-fed and bottle-fed infants, it was found that serum BPA was detected in both groups. This suggests that developmental exposure to BPA is almost ubiquitous [14]. Furthermore, exposure throughout life is likely to be continuous, and it is difficult to make conclusions about the time of BPA exposure. This problem affects most data examined relationship between exposure of BPA and a lot of diseases [41]. We did not assess the variability of exposure over time, but most data assumed that this exposure is continuous (living environment, consumption habits, and exposure source) [26].

Many studies proved that the perinatal period is a sensitive window of exposure to BPA in the male animal reproductive system [23]. According to Fiorini, Sertoli cell tight junction proteins are early targets for BPA and other testicular toxicants. BPA affects Sertoli cell tight junction proteins by either reducing the amount or inducing aberrant intracellular localization of these membranous proteins. A consequence of this is an impaired process of spermatogenesis, which is controlled by endocrine and local intercellular communications mediators [42].

The human placenta does not function as a barrier to maternal BPA [43]. Damgaard et al. found a correlation between cryptorchidism and the presence of some persistent pesticides in breast milk. Their study suggests that exposure to more than one chemical at low doses represents a risk factor for congenital cryptorchidism [44]. Contrary to this observation, Virtanen et al. found no relationship between dioxins and bisphenols in the placenta and cases of cryptorchidism [45]. Also Hosie et al. did not show statistical differences in the level of BPA between boys undergoing orchiopexy and a control group [46].

On the contrary, in a study from France, median PCB levels (polychlorinated biphenyls) tended to be higher in breast milk, but not in cord serum, in boys with cryptorchidism [47]. In our study, total and conjugated serum BPA levels were higher in boys with cryptorchidism than in the control group. The difference was statistically significant. We believe that our observation reflects the continuous exposure to BPA in our patients, connected with environmental sources. On the other hand, recent studies have shown that uBPA levels were similar in boys with or without undescended testis [48]. Chevalier et al. found that cord blood levels of BPA correlated negatively with INSL3. The authors found that levels of INLS3 were significantly decreased in boys with undescended testis, while levels of BPA in cord blood were increased but not significantly. They suggested that during fetal testis development INSL3 could be a possible target of EDCs. Additionally, comparing boys with palpable and nonpalpable undescended testis, the levels of BPA in cord blood were higher, though not significantly, in the second group [49].

Experiments on animals suggested that prenatal and early postnatal exposure to BPA adversely affected spermatogenesis and sperm quality [50]. Human studies on the effect of adult exposure to BPA on sperm quality are limited and results are contradictory. In some studies, higher urinary BPA levels were associated with a decrease in sperm count and motility [51, 52]. Others studies did not show this correlation, although the markers of free testosterone level were modestly reduced [53]. A study on rats showed that continuous exposure to high doses of BPA decreased the number of Leydig cells and the expression of steroidogenic enzymes [54].

## 5. Conclusions

Our study indicated that in boys with cryptorchidism total and conjugated serum BPA level was higher. We believe that our observation reflects the continuous exposure to BPA in our patients, connected with environmental sources. Prospective and long-term follow-up studies should be conducted to examine relationship between BPA levels and cryptorchidism. Multiple collections from children would be more useful to determine potential cause-and-effect relationship.

## Figures and Tables

**Table 1:** Serum BPA level in cryptorchid and control group.

	Control group (*n* = 57)	Cryptorchid (*n* = 98)	*p* value
Serum free BPA level (ng/mL)	5.7 (0.05–83.4)	5.3 (0.11–112.1)	*p* > 0.05 (*p* = 0.818)
Serum conjugated BPA level (ng/mL)	11.4 (0.0–56.7)	15.8 (0.0–83.1)	*p* ≤ 0.05 (*p* = 0.502)
Serum total BPA level (ng/mL)	16.1 (4.5–68.5)	25.4 (4.1–89.8)	*p* < 0.05 (*p* = 0.0461)

**Table 2:** Parents' answers to selected questions from the risk of environmental bisphenol exposure questionnaire. Explanation of abbreviations used in the table is in the first column.

	Cryptorchid (*n* = 98)	Control (*n* = 57)
Age (months)	27 (10–48)	27 (10–48)
X-ray exams: 1 or more during life	26.3%	28.1%
Place of living (village/city)	v-43.2%; c-56.8%	v-42.1%; c-57.9%
Pregnancy complications (yes/no)	y-10.5%; n-89.5%	y-14.5%; n-85.5%
Hormonal treatment of a child (yes/no)	y-3.2%; n-96.8%	y-0%; n-100%
Problem with conception (yes/no)	y-7.4%; n-92.6%	y-7.0%; n-93%
Hormonal treatment before the pregnancy (yes/no)	y-11.1%; n-80.9%	y-29.8%; n-70.2%
Cigarettes smoking, mother (yes/no)	y-20.0%; n-80%	y-22.8%; n-77.2%
Testicular cancer in close family (yes/no)	y-2.1%; n-97.9%	y-5.3%; n-94.7%
Consumption of smoked products (frequently/rare/never)	f-25.3%; r-68.4%; n-6.4%	f-31.6%; r-59.6%; n-8.8%
Consumption of foods packed with a long expiration date (frequently/rare/never)	f-9.5%; r-70.5%; n-20.0%	f-8.8%; r-75.4%; n-15.8%
Consumption of drinks in plastic packaging (frequently/rare/never)	f-53.7%; r-37.9%; n-8.5%	f-50.9%; r-42.1%; n-7.0%
